# The Maillard Reaction as Source of Meat Flavor Compounds in Dry Cured Meat Model Systems under Mild Temperature Conditions

**DOI:** 10.3390/molecules26010223

**Published:** 2021-01-04

**Authors:** Lei Li, Carmela Belloch, Mónica Flores

**Affiliations:** Instituto de Agroquímica y Tecnología de Alimentos (IATA-CSIC), Avda. Agustín Escardino 7, 46980 Paterna, Valencia, Spain; leili@iata.csic.es (L.L.); belloch@iata.csic.es (C.B.)

**Keywords:** meat flavor, dry cured, mild conditions

## Abstract

Flavor is amongst the major personal satisfaction indicators for meat products. The aroma of dry cured meat products is generated under specific conditions such as long ripening periods and mild temperatures. In these conditions, the contribution of Maillard reactions to the generation of the dry cured flavor is unknown. The main purpose of this study was to examine mild curing conditions such as temperature, pH and a_w_ for the generation of volatile compounds responsible for the cured meat aroma in model systems simulating dry fermented sausages. The different conditions were tested in model systems resembling dry fermented sausages at different stages of production. Three conditions of model system, labeled initial (I), 1st drying (1D) and 2nd drying (2D) and containing different concentrations of amino acid and curing additives, as well as different pH and a_w_ values, were incubated at different temperatures. Changes in the profile of the volatile compounds were investigated by solid phase microextraction and gas chromatography mass spectrometry (SPME-GS-MS) as well as the amino acid content. Seventeen volatile compounds were identified and quantified in the model systems. A significant production of branched chain volatile compounds, sulfur, furans, pyrazines and heterocyclic volatile compounds were detected in the model systems. At the drying stages, temperature was the main factor affecting volatile production, followed by amino acid concentration and a_w_. This research demonstrates that at the mild curing conditions used to produce dry cured meat product volatile compounds are generated via the Maillard reaction from free amino acids. Moreover, in these conditions a_w_ plays an important role promoting formation of flavor compounds.

## 1. Introduction

Flavor is among the most important quality indicators of meat products. Flavor compounds produced during the processing of meat products are generated through different mechanisms including the Maillard reaction, Strecker degradation, lipid oxidative reactions, degradation of thiamine, ribonucleotides and carbohydrates, as well as microbial metabolism [1,2]. The contribution of these reactions to meat flavor depends on the manufacture conditions applied during processing.

Flavor compounds present in cooked cured meat products have been studied qualitatively and quantitatively [3], the Maillard reaction being the most important reaction for the production of meaty aroma compounds [4]. The Maillard reaction between an amino group and a reducing sugar is commonly divided into three stages: Amadori/Heyns rearrangement, sugar fragmentation and retro-aldolization. In these reactions the free amino group of amino acids peptides or proteins participates in dehydration, fragmentation, cyclization and polymerization reactions. The different paths involved in the Maillard reaction depend on conditions such as temperature, pH and water content that regulate the reaction kinetics, and type of sugar and amino acids that modulate the flavor compounds formed [5]. Extensive investigation of flavor formation in cooked cured meat products [6] has revealed the effect of cooking temperature, amino acid and sugar types and lipid composition [7]. Meaty aroma seems to be constituted by different volatile compounds, although the contribution of sulfur-containing compounds is essential [1]. Among these, thiophenes are derived mainly from the Maillard reaction between cysteine and ribose [5]. Amino acids such as cysteine and glycine are of utmost importance as precursors of sulfur-containing meaty compounds [8,9]. Furthermore, meat post-mortem conditioning increases the formation of Maillard reaction-derived meaty flavor compounds by the presence of ribose, methionine and cysteine [10].

Few studies have focused on the role of Maillard kinetics for flavor generation during dry cured meat processing. In these types of products, low temperatures are applied during long processing times depending on products, ranging between 2 to 3 months in dry fermented sausages, and up to 2–3 years in dry cured hams [11]. Flavor production during dry cured meat processing is influenced by the use salt and nitrate and/or nitrite, which are rubbed on the surface of meat or mixed with the minced meat. As a result of the proteolysis occurring during dry curing, there is an increase in free amino acids which are flavor precursors in dry cured meat products [12]. Both pH and a_w_ exert a profound influence on the abundance of meaty flavor compounds in thermal treatments [13]. However, flavor composition of dry cured meat products and cooked cured meat products is significantly different due to the different processing methods [1]. The meaty flavor compounds of dry cured meat products are produced under long ripening periods where mild temperatures are applied.

The mechanisms involved in the generation of meaty flavor compounds in dry fermented sausages and the influence of the physicochemical conditions such as pH and low temperature are poorly understood, whereas many studies have focused on the microbial contribution during fermentation [14,15]. Additionally, several studies have focused on the lipid oxidation effect on dry sausage flavor [16]. However, the role of Maillard reactions in dry sausage flavor generation has not been elucidated. The control of flavor formation in meat products from Maillard reactions is essential for flavor quality [17]. Therefore, new studies on Maillard reactions are necessary to identify the influence of critical process parameters (pH, temperature, a_w_) as well as determine the effect of reactant concentrations on the formation of flavor compounds in dry cured meat products.

The main purpose of this study was to examine mild curing conditions as far as temperature, pH and a_w_ for the generation of volatile compounds responsible for the cured meat aroma in model systems simulating dry fermented sausages. Model systems characterized by different amino acid and additive composition were incubated at 25 and 37 °C and samples taken at different times for profiling of generated volatile compounds and amino acid content.

## 2. Results

### 2.1. Analysis of Physicochemical Changes in the Model Systems

Water activity (a_w_) and pH (Figures S1 and S2 Supplementary Material, respectively) were measured during the incubation of sausage model systems (media I, 1D and 2D) at mild conditions (25 and 37 °C), and both parameters remained stable from the first day of incubation until 35 d.

### 2.2. Identification of Volatile Compounds Generated in the Model Systems

The identification of volatile compounds generated in the model systems is listed in Table 1. Seventeen volatile compounds were identified, and 1 of them (furan) was only tentatively identified. The compounds identified belonged to different chemical classes: 2 ketones, 2 furans, 6 aldehydes, 1 sulfur compounds, 1 pyrazine, 1 alcohol, 1 acid and 3 hydrocarbons. Not all compounds were detected in the three models. Furan, dimethyl disulfide, thiazole and methyl-pyrazine were absent in model I at both temperature conditions. Additionally, furfural was only detected when the three models were incubated at 37 °C. The volatile compounds detected were mainly derived from branched chain amino acids (Val, Leu and Ile); sulfur amino acids (Met and Cys); and Phe, Ala, Thr and Ser.

### 2.3. Quantitation of Volatile Compounds Generated in the Model Systems

**Figure 1:**
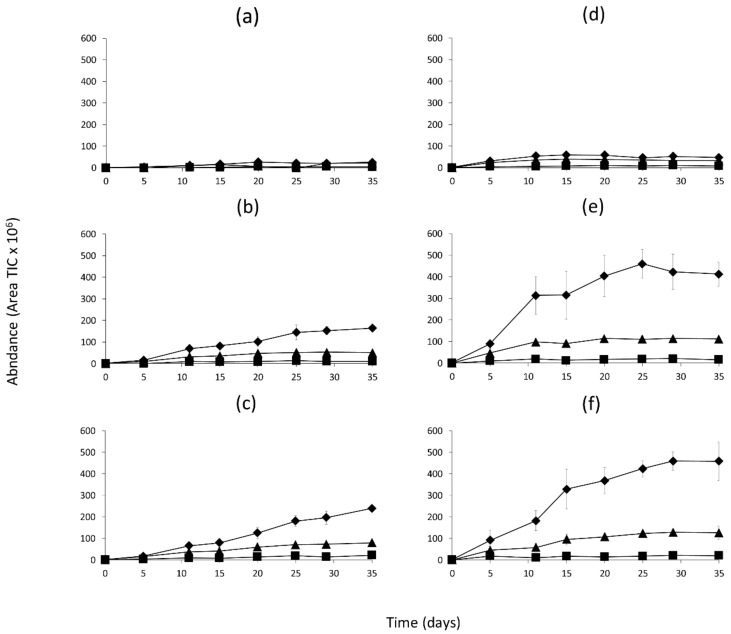
Abundance of volatile compounds derived from Val, Ile and Leu in the model systems during incubation at 25 or 37 °C and classified according to the dry curing stages (I-Initial stage, 1D-1st drying stage, 2D-2nd drying stage). (**a**) I-25 °C, (**b**) 1D-25 °C, (**c**) 2D-25 °C, (**d**) I-37 °C, (**e**) 1D-37 °C, (**f**) 2D-37 °C. Symbols represent the different compounds: 2-Methylpropanal (■); 3-Methylbutanal (♦); 2-Methylbutanal (▲).

**Figure 2:**
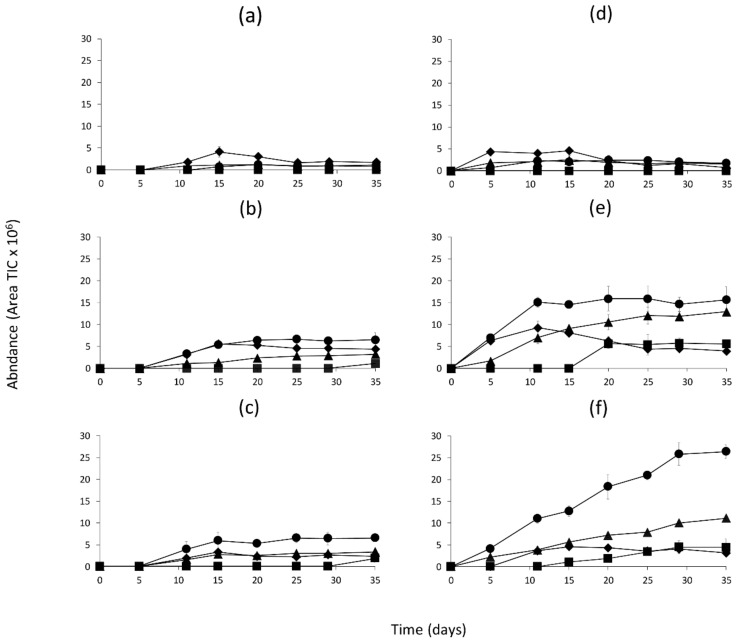
Abundance of volatile compounds derived from sulfur amino acids and Phe in the model systems during incubation at 25 or 37 °C and classified according to the dry curing stages (I-Initial stage, 1D-1st drying stage, 2D-2nd drying stage). (**a**) I-25 °C, (**b**) 1D-25 °C, (**c**) 2D-25 °C, (**d**) I-37 °C, (**e**) 1D-37 °C, (**f**) 2D-37 °C. Symbols represent the different compounds: Dimethyl disulfide (■); 3-(Methylthio)propanal (♦); Benzaldehyde (▲); Benzeneacetaldehyde (●).

**Figure 3:**
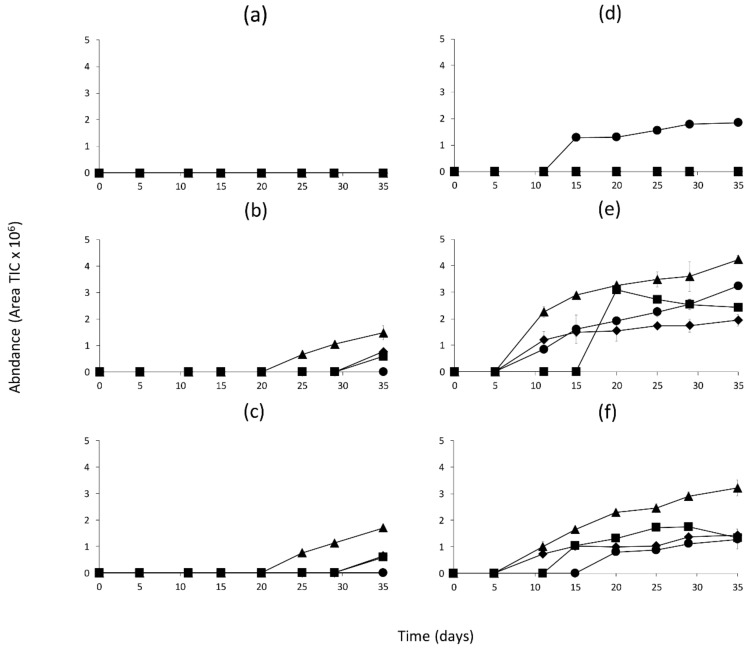
Abundance of heterocyclic volatile compounds derived from other amino acids in the model systems during incubation at 25 or 37 °C and classified according to the dry curing stages (I-Initial stage, 1D-1st drying stage, 2D-2nd drying stage). (**a**) I-25 °C, (**b**) 1D-25 °C, (**c**) 2D-25 °C, (**d**) I-37 °C, (**e**) 1D-37 °C, (**f**) 2D-37 °C. Symbols represent the different compound: Furan (■); Thiazole (♦); Methylpyrazine (▲); Furfural (●).

The quantitation of all the volatile compounds generated in the model systems incubated at 25 and 37 °C is shown in Tables S1 and S2 of Supplementary Material. Figure 1, Figure 2 and Figure 3 show the changes in abundance of the most variable volatile compounds derived from branched-chain amino acids Val, Leu and Ile (Figure 1); sulfur amino acids (Met and Cys) and Phe (Figure 2); and heterocyclic compounds (Figure 3).

Figure 1 shows that model system I (Figure 1a,d) was very poor in volatile compounds derived from Val, Ile and Leu at both incubation temperatures and any time. Model 1D (Figure 1b,e) showed a significant (*p* < 0.001) increase in the content of 3-methylbutanal during the incubation time. This increase was even higher when the model system was incubated at 37 °C. A similar trend was also observed in the case of 2-methylbutanal, although this compound was produced in lower amounts. In model 2D (Figure 1c,f), the evolution of 3-methylbutanal and 2-methylbutanal was similar than in model 1D, but the changes were significantly higher (*p* < 0.001) when the model system was incubated at 37 °C.

In Figure 2, the changes observed in the volatile compounds in model system I (Figure 2a,d) were not significant at any incubation temperature or time. On the contrary, model systems 1D and 2D showed a significant (*p* < 0.001) increase in benzaldehyde and benzeneacetaldehyde during the incubation time and, as in Figure 1, this increase was significantly higher at 37 °C (Figure 2b,c vs. 2e,f). Dimethyl disulfide was detected depending on the temperature and time of incubation. At 25 °C, it was detected at the end of incubation time (35 d) (Figure 2b,c), whilst at 37 °C, it was detected at 15 (2D) or 20 d (1D) of incubation time (Figure 2e,f). The abundance of this compound increased with the incubation time. Moreover, 3-(methythio)propanal was detected in higher abundance at 37° (Figure 2f) than at 25 °C (Figure 2b,c), although in both conditions it increased until 10–15 d but showed a small decrease afterwards.

In Figure 3, we can observe an increase in furfural in model system I at 37 °C (Figure 3a,d). In model systems 1D and 2D (Figure 3b,e; Figure 3c,f), methylpyrazine, thiazole, furan and furfural increased significantly (*p* < 0.001) during the incubation time, although this increase was significantly higher when the models were incubated at 37 °C.

**Figure 4:**
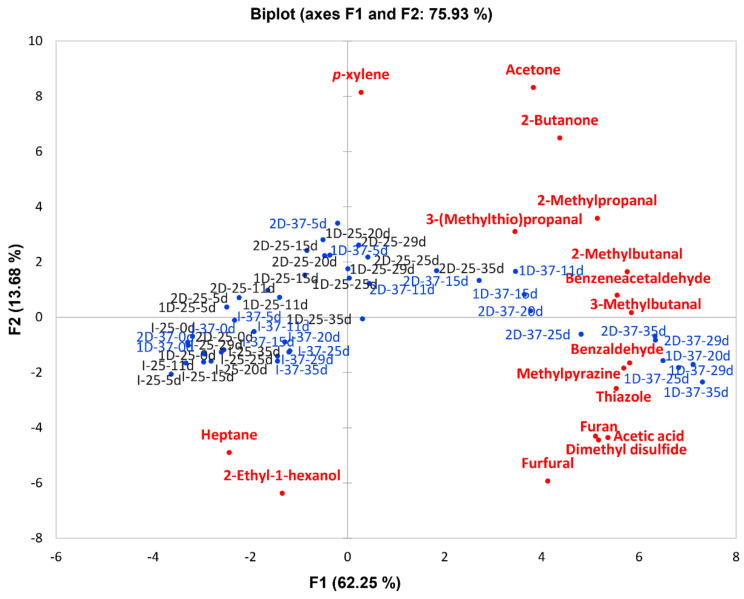
Loadings of the first two principal components (PC1-PC2) representing the variability (volatile compounds) in the model systems simulating the dry curing stages (I-Initial stage, 1D-1st drying stage, 2D-2nd drying stage). Sample loadings in black and blue represent models incubated at 25 and 37 °C, respectively.

In order to examine the relationship between the different volatile profiles in the model systems under mild conditions (25 and 37 °C) and the flavor compounds produced, a principal component analysis (PCA) was performed (Figure 4). Two principal components were able to explain the 75.93% of the total variability. PC1 accounts for 62.25% of the variability and was strongly related with the incubation time. Model I, characterized by short incubation times, was located on the left quadrant, while model systems 1D and 2D, incubated for longer times, were on the right. This would indicate that PC1 takes into account model system composition and incubation time. PC2 accounts for 13.88% of the variability and distinguishes samples by different groups of flavor compounds. Most of the aldehydes and ketones were on the upper quadrants, whereas on the lower quadrants, furans (furan and furfural), cyclic compounds (methylpyrazine and thiazole) and phenyl compounds (benzaldehyde) were related to models 1D and 2D incubated at 37 °C during longer times.

### 2.4. Amino Acid Concentration in the Model Systems

**Figure 5:**
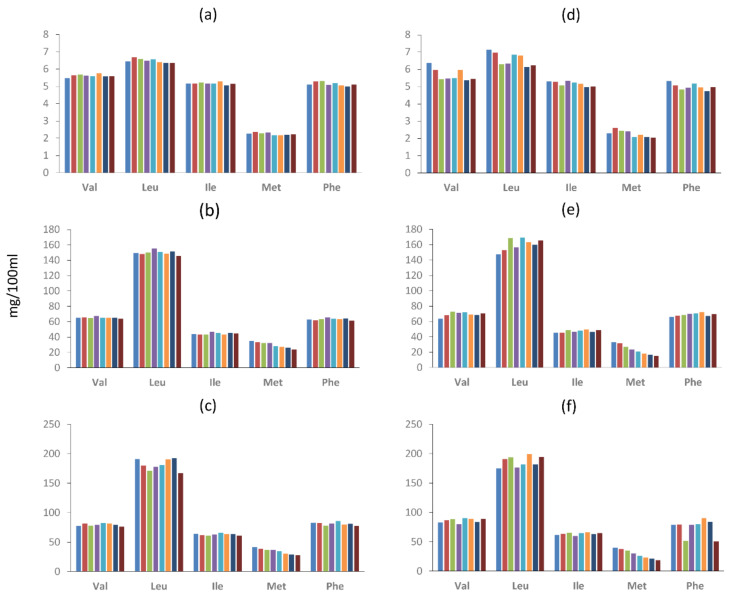
Amino acid concentration in the model systems (mg/100 mL) during incubation at 25 or 37 °C and classified according to the dry curing stages (I-Initial stage, 1D-1st drying stage, 2D-2nd drying stage). (**a**) I-25 °C, (**b**) 1D-25 °C, (**c**) 2D-25 °C, (**d**) I-37 °C, (**e**) 1D-37 °C, (**f**) 2D-37 °C. Bars from right to left in each amino acid represent concentrations at 0, 5, 9, 11 until 35 d.

The concentration of amino acids was compared among the model systems during the incubation time (Table S3 Supplementary Material). Due to limitations of the analysis method, two amino acids, Arg and Cys, were not analyzed. The concentrations of the amino acids (Val, Leu, Ile, Pro, Met, Phe and orn) during the incubation times (0 to 35 d) and temperatures (25 and 37°) in the different models (I, 1D and 2D) are shown in Figure 5. Amino acid concentration in model system I did not show significant differences due to incubation times at any temperatures 25 or 37 °C (Figure 5a,d). On the other hand, a significant (*p* < 0.001) decrease in met during the incubation time was observed in models 1D and 2 D (Figure 5b,c and Figure 5e,f) at both temperatures.

## 3. Discussion

Flavor formation in dry cured meat products is very complex because many processing conditions affect its generation [1]. For example, Zamora and Hidalgo [27] indicated the importance of lipid oxidation and Maillard reactions in food processing, and indicated that both reactions are interrelated and the products of each reaction can modify the other.

The results from our experiments demonstrated that model systems that simulate the intermediate processing stages of dry fermented sausages, 1D and 2D, have different volatile profiles than the model simulating the initial conditions (model I) (Table 2). The conditions in model I were not sufficient to promote Maillard reactions and formation of volatile compounds, because only furfural was detected at 37 °C (Figure 3). This would indicate that whilst amino acids were present in model I, higher concentrations of amino acids (10 times more) are necessary for volatile generation, as occurs in models 1D and 2 D (Figure 5 and Table S3). Therefore, the proteolytic activity occurring during the drying process is essential to the release of free amino acids [12,14] and generation of Maillard reaction products under mild conditions. Moreover, the results showed that formation of Maillard volatile compounds in models 1D and 2D was compound-specific and dependent on the physicochemical conditions (pH, a_w_, temperature).

The compounds derived from Val, Leu and Ile were produced in 1D and 2D models and dependent on the concentration of these amino acids in the models. The compound produced in the highest quantity 3-methylbutanal was derived from Leu, which was at the highest concentration (Figure 5). Compound 2-methylpropanal appeared always in low amounts (Figure 1) and was not affected by the mild conditions (pH, a_w_ and temperature) or concentration of precursor Val (Figure 5). The most probable explanation for the generation of this compound in fermented sausages is its production by the activity of Micrococcaceae such as *Staphylococcus* [28] and not from Maillard reactions. The increase in 3-methylbutanal with the incubation time in models 1D and 2D, which could be related to the amino acid content and favored by the lowest a_w_ in 2D (Figure S1). Previous studies have pointed out that Strecker aldehydes reach a maximum at high temperatures because they are not end products and, therefore, can react further to form other compounds [29]. Similarly, 3-methylbutanal in model 1D at 37 °C reached its highest value at 25 days and afterwards decreased. Moreover, a_w_ seems to be an essential parameter in Maillard reactions [30], as maximum reaction rates were found at 0.6–0.8 a_w_ values [31]. These branched aldehydes have been identified as key aroma compounds in dry-cured fermented sausages due to their aroma notes and low thresholds (Table 1) [32]. Similar dynamics were found for 2-methylbutanal generation in models 1D and 2D; however, the low Ile concentration (Figure 5) would have prevented higher production of this compound. In summary, a high amino acid content favored the production of branched aldehydes, the production of which further increased under low a_w_. On the other hand, pH was revealed as an important factor in Maillard reactions [17], affecting the volatile compounds generated [22] even at the small differences observed between 1D and 2D models (0.2 units).

Dimethyl disulfide and 3-(methylthio)propanal generation was related to the Strecker degradation of the amino acid Met (Table 1). The dynamic of 3-(methylthio)propanal generation was different from other volatile compounds produced in the three models. The concentration of this compound increased with incubation time until 10 to 15 d and then decreased specially in 1D followed by 2D and I model systems. This decrease could be related to an oxidative chemical reaction from l-methionine as observed in other studies [33]. Met in models 1D and 2D favored the generation of 3-(methylthio)propanal versus dimethyl disulfide (Figure 2e,f). Met content was very similar in 1D and 2D (Figure 5), showing a significant decrease during incubation which was more pronounced at 37 °C, in agreement with the higher generation of volatiles at this temperature (Figure 5). This demonstrated that temperature was a key factor in the formation of volatile compounds from Met, but the low temperature applied during dry cured meat processing (ranging from 10 to 25 °C depending on the ripening stage [11]) does not favor their generation. Regarding pH and a_w_ conditions, model 1D at lower pH and higher a_w_ than 2D favored the formation of dimethyl disulfide and 3-(methylthio)propanal. Therefore, the lowest pH values in 1D seemed to favor formation of these compounds, as observed in Maillard models submitted to high cooking temperatures [22]. Additionally, Phe concentration in models 1D and 2D together with 37 °C temperature seemed to be the most important factor for volatile production, although the lower a_w_ in 2D (Figure S1) produced a significant effect.

Furan is generated by two major formation pathways: (1) the intact sugar skeleton and (2) recombination of reactive C2 and/or C3 fragments which come from the amino acids Ala, Thr or Ser [18]. In the model systems, furan formation was specially favored by high temperature as observed in models incubated at 37 °C. Concentration of Ala and Thr was higher in 2D than 1D, although the higher Ser content in 1D may have favored furan production in this model (Table S3 Supplementary Material). Furfural appeared to be an intermediate of the Maillard reaction, and its concentration increased with temperature and time (Figure 3). Furfural was the only compound detected in I model, which contained the highest glucose concentration (Table 3). Furfural has been detected as a major product in model systems related to sugar degradation in Maillard reactions and its formation has been related with low pH values (pH < 7.0) [31]. In models 1D and 2D, where glucose was low in comparison to I model, furfural formation was favored by the lowest pH (4.3) value in 1D model.

Generation of methylpyrazine exclusively in 2D and 1D at 25 and 37 °C was favored by the presence of Lys and high pH values [22,23]. Methylpyrazine content was the highest in model 1D, although Lys was in higher quantity in model 2D. This would indicate that in addition to Lys other factors affect the generation of this compound.

Other volatile compounds produced in the model systems such as acetic acid and thiazole were also produced in higher quantities in models incubated at 37 °C than at 25 °C (Table S3 Supplementary Material). Acetic acid (Table 1), an indicator of the progress of Maillard reaction [34], was identified as byproduct of sugar degradation [18]. Thiazole, a reaction product between cysteine and carbonyls [21], was only generated in models 2D and 1D at 37 °C; therefore, temperature seems to be a key factor in the formation of this compound.

This study demonstrates that mild processing conditions such as low temperatures (25 and 37 °C), pH and a_w_ can be responsible for the different volatile profiles found in the model systems. These mild conditions applied during dry curing show that different aroma compounds can be generated by α-dicarbonyl compounds and amino acids as reported in wine aging [35]. In dry meat products, Zhu [36] evaluated the reactions between glucose and acetaldehyde with histidine and lysine in the generation of meat flavor compounds under mild conditions. These authors observed that histidine and lysine were key precursors of Maillard reaction products that constitute the specific flavor of Jinhua ham. The results obtained with the model systems revealed a higher number of volatile compounds such as pyrazines, furan and sulfur compounds, not found in previous experiments [36] due to the limited number of amino acids and carbonyl compounds.

Another important result of our study is that high glucose content seems not essential for the generation of Maillard reaction volatile compounds, because the four times higher concentration of this compound in model I with respect to models 1D and 2D only resulted in the generation of furfural. Sugar content in dry fermented products decreases during the fermentation stage as it is used by the microorganisms to produce a decline in pH that favors sausage drying. However, although the nature and content of saccharides has been described as essential in Maillard reactions [31], in dry meat processes, it seems that the limiting factor to produce volatile compounds from Maillard reactions is the amino acid content as well as temperature and a_w_ conditions.

The contribution of the volatile compounds to the aroma of the dry cured products is a balance among all the volatile compounds identified in the product [1]. Nevertheless, taking into consideration that the compounds contributing to the aroma, such as 2-methylpropanal, 2 and 3-methylbutanal, 3-(methylthio)propanal, benzaldehyde, benzeneacetaldehyde, dimethyl disulfide and thiazole (Table 1), have the lowest threshold values in oil, they can be considered important contributors to the aroma in dry sausages [37,38,39]. Moreover, generation of these aroma compounds was mainly observed at long incubation times at mild temperatures in models 1D and 2D indicating the key impact of temperature on aroma formation in dry meat processes.

## 4. Materials and Methods

The different dry cured meat model systems were prepared according to the dry fermented sausages composition [40,41] regarding the concentration of additives and free amino acids at the initial (I), 1st drying (1D) and 2nd drying (2D) stages of the process (Table 2). Model systems were prepared in 0.2 mM phosphate buffer, and their composition included nitrate, nitrite, NaCl, sodium ascorbate and glucose at the concentrations included in Table 3. a_w_ of the model systems at the different processing stages was adjusted with glycerol. Sterilization was performed by filtration using a 0.2 μm filter (Sartorius, Göttingen, Germany), and incubation of the three model systems was performed at 25 and 37 °C. The experiments were performed in triplicate. Samples from each media and temperature were taken at 0, 5, 11, 15, 20, 25, 29 and 35 days for physicochemical analysis, volatiles and amino acid composition.

### 4.1. Analysis of Water Activity and pH

Water activity (a_w_) was measured using an AQUALAB^®^ 4 Water Activity Meter (METER Group, München, Germany), and pH was measured using a pH-meter (Orion EA 920, Boston, MA, USA).

### 4.2. Analysis of Volatile Compounds

Analysis of volatile compounds was carried out by headspace (HS) solid-phase microextraction (SPME) with an 85 μm carboxen/polydimethylsiloxane (CAR/PDMS) fiber (Supelco, Bellefonte, PA, USA) using a gas chromatograph (Agilent HP 7890 series II, Hewlett-Packard, Palo Alto, CA, USA) with a quadrupole mass detector (HP 5975C, Hewlett-Packard, Palo Alto, CA, USA). In summary, 5 mL of the model system was placed into a headspace vial using an automatic injector Gerstel MPS2 (Gerstel, Germany) [15] and incubated at 37 °C for 30 min. The extracted volatile compounds were adsorbed in the fiber for 60 min at 30 °C and desorbed in the injection port of the GC–MS for 5 min at 240 °C in splitless mode. The volatile compounds were separated using a DB-624 capillary column (30 m, 0.25 mm i.d., film thickness 1.4 μm (J&W Scientific, Agilent Technologies, Palo Alto, CA, USA)) using the conditions described by Corral et al. [42]. The MS interface temperature was set to 240 °C. The compounds were identified in full scan mode and by comparison with mass spectra from the library database (Nist’05), with linear retention indices [43] and using authentic standards. The quantitation was performed in SCAN mode using either total ion current (TIC) on an arbitrary scale and expressed as abundance units (AU) × 10^6^.

### 4.3. Analysis of Amino Acids

The analysis of free amino acids was performed using the EZ-Faast kit from Phenomenex (Torrance, CA, USA). Model system samples were diluted at ratio 1:5 (*v*/*v*) for I stage, and 1:25 (*v*/*v*) for 1D and 2D stages with distilled water previously to derivatization. The derived amino acids were analyzed using GC-FID. A gas chromatograph (Agilent Technologies 7890B) with a flame ionization detector (FID) equipped with an autosampler G4513A and a ZB-AAA 10 m × 0.25 mm GC column (Phenomenex) was used. The injection volume was 2.5 μL at 250 °C in split mode (15:1). Helium was used as a carrier gas at a constant flow of 27 mL/min during the run, and the column head pressure was 8.78 psi. The GC oven temperature was initially held at 110 °C and then raised to 320 °C at 32 °C/min; the inlet temperature was 250 °C, and the detector was set at 320 °C. Identification and quantification were based on retention time and peak area integration of the reference amino acids. Norleucine was used as the internal standard. Calibration curves for each amino acid were obtained with the standard amino acids solutions (Phenomenex). In addition, several amino acids were prepared at high concentrations to adjust the calibration curves to the levels present in media samples. Results were expressed in milligrams per 100 mL of model system.

### 4.4. Statistical Analyses

The effect of different model system composition and incubation time on the generation of volatile compounds were tested by two-factor analysis of variance (ANOVA) at each temperature (25 and 37 °C) using the statistic software XLSTAT2018 (Addinsoft, Barcelona, Spain). The effect of temperature and time on the amino acid content was studied in each media by a two-factor analysis of variance (ANOVA). Significant differences between samples means were analyzed according to Tukey test (*p* < 0.05). Principal component analysis (PCA) was plotted to evaluate the relationships among the different ripening stages, incubation temperature and volatile compounds in model systems measured at each sampling time.

## 5. Conclusions

This study has shown high yields of volatile compounds produced in model systems resembling dry fermented meat products at different curing stages in terms of amino acid content, pH, lower a_w_ and temperature (37 vs. 25 °C). In order to improve the formation of aroma compounds during the ripening process, the increase in temperature during dry curing process would favor their generation once the a_w_ value is low and amino acid concentration increases from the proteolytic activity. This research demonstrates that in dry fermented meat products, free amino acids participate in the generation of volatile compounds via the Maillard reaction in addition to the microbial activity. Moreover, a_w_ and temperature play an important role in these processes promoting the formation of flavor compounds in dry cured meat products.

## Figures and Tables

**Table 1:** Volatile compounds identified in the model systems.

Compound	LRI ^1^	LRI std ^2^	RI ^3^	Initial Stage		1st Drying Stage		2nd Drying Stage		Aac Precursor ^5^	Odor ^6^	Threshold in Oil (mg/kg) ^7^
				I-25°	I-37°	1D-25°	1D-37°	2D-25°	2D-37°			
Furan	514		b			+ ^4^	+	+	+	Ala, Thr, Ser [18]	ethereal	4.5–6 ^a^
Acetone	530	527	a	+	+	+	+	+	+		ethereal	100–1000
2-Methylpropanal	594	590	a	+	+	+	+	+	+	Val [19]	pungent, floral	0.043
2-Butanone	632	629	a		+	+	+	+	+		woody, yogurt	10–100
3-Methylbutanal	692	687	a	+	+	+	+	+	+	Leu [19]	fruity green cocoa	0.0054–0.013
Heptane	700	700	a	+	+	+	+	+	+		-	
2-Methylbutanal	702	698	a	+	+	+	+	+	+	Ile [19]	musty chocolate, malty	0.0022–0.152
Acetic acid	715	714	a	+	+	+	+	+	+		pungent vinegar	0.12–0.75
Dimethyl disulfide	773	774	a			+	+	+	+	Met [20]	onion, cabbage	0.012
Thiazole	776	776	a			+	+	+	+	Cys [21]	nutty, meaty	0.038–3.1 ^a^
Methylpyrazine	860	860	a			+	+	+	+	Gly, Lys, Cys [22,23]	nutty, cocoa, roasted,	27
*p*-xylene	892	893	a	+	+	+	+	+	+		*-*	
Furfural	898	898	a		+		+		+		brown, bready, nutty, caramel	155 ^b^
3-(Methylthio)propanal	968	968	a	+	+	+	+	+	+	Met [24]	cooked potato, onion,	0.0002
Benzaldehyde	1020	1013	a	+	+	+	+	+	+	Phe [25]	bitter almond, cherry	0.06
2-Ethyl-1-hexanol	1083	1083	a	+	+	+	+	+	+		sweet, fatty, fruity	0.27–25 ^a^
Benzeneacetaldehyde	1110	1104	a	+	+	+	+	+	+	Phe [26]	floral, chocolate	0.002–0.03 ^a^

^1^ LRI: Linear retention indices of the compounds eluted from the GC-MS using a DB-624 capillary column. ^2^ LRI std: Linear Retention Indices of authentic standard compounds. ^3^ RI: Reliability of identification: a: identification by mass spectrum and coincident with LRI of an authentic standard; b: tentative identification by mass spectrum. ^4^ Blank space means not generated; + indicates the compound was detected. ^5^ Bibliographic references indicating the amino acid precursor of the volatile compound are between brackets. ^6^ Burdok, G. A. (2010). Fenaroli’s handbook of flavor ingredients (6th ed.). Florida: Boca Raton. CRC Press Inc. ^7^ Van Gemert, L., and Nettenbreijer, A. (2011). Compilation of odor threshold values in air, water and other media. The Netherlands: BACIS: Zeist. ^a^ Threshold reported in water; ^b^ threshold reported in propylenglycol.

**Table 2:** Composition in amino acids (mg/100 mL) of each model system according to the concentration of amino acids reported in different ripening stages of dry fermented sausages [40,41].

Amino Acids	Initial Stage	1st Drying Stage	2nd Drying Stage
Ala	29.91	91.85	111.80
Gly	10.94	33.06	42.58
Val	6.01	72.05	90.35
β-Ala	3.48	4.35	4.54
Leu	5.96	151.80	176.80
Ile	4.17	45.49	62.08
Thr	4.55	35.15	54.99
Ser	4.72	31.96	19.24
Pro	4.26	83.60	89.05
Asn	1.40	14.74	21.71
Asp	16.67	106.70	100.10
Met	2.00	33.99	36.27
Glu	10.53	74.25	73.45
Phe	3.82	70.95	83.85
Gln	36.47	64.90	65.00
Orn	0.23	23.10	31.01
Lys	6.82	77.55	120.90
His	2.76	27.34	38.48
Tyr	4.47	29.21	26.98
Trp	1.34	16.89	17.03
C-C	2.53	3.22	5.36
Arg	8.30	5.29	9.49
Cys	4.03	10.40	11.12

**Table 3:** Physicochemical conditions of the model systems simulating the ripening stages in the production of dry fermented sausages.

Dry Curing Stages	Initial Stage	1st Drying Stage	2nd Drying Stage
pH	5.45	4.38	4.60
Glycerol (ml/100 mL)	0	9	23
a_w_	0.968	0.944	0.895
NaCl (mg/mL)	27	27	27
NaNO_2_ (mg/mL)	0.15	0	0
NaNO_3_ (mg/mL)	0.15	0.10	0.075
Sodium ascorbate (mg/mL)	0.5	0.5	0.5
Glucose (mg/mL)	20	5	5
Amino acids (mg/100 mL)	Table 2 (Initial stage)	Table 2 (1st drying stage)	Table 2 (2nd drying stage)

## Data Availability

Data is contained within the article or supplementary material.

## Supplementary Materials

The following are available online. Figure S1: Changes in aw of the model systems during incubation at 25 or 37 °C simulating the dry curing stages (I-Initial stage, 1D-1st drying stage, 2D-2nd drying stage). Figure S2: Changes in pH of the model systems during incubation at 25 or 37 °C simulating the dry curing stages (I-Initial stage, 1D-1st drying stage, 2D-2nd drying stage). Table S1: Volatile compounds produced at 25 °C in the model systems simulating the dry curing stages (expressed as abundance (AU) of total ion current (TIC) as AU × 106). Table S2: Volatile compounds produced at 37 °C in the model systems simulating the dry curing stages (expressed as abundance (AU) of total ion current (TIC) as AU × 106). Table S3: Concentration of amino acids (mg/100 mL) in the model systems simulating the dry curing stages.

## Sample Availability

Samples of the compounds are available from the authors.
