# Supplementary figures and images for: The Maillard Reaction as Source of Meat Flavor Compounds in Dry Cured Meat Model Systems under Mild Temperature Conditions
Source: Molecules. 2021 Jan 4;26(1):223. doi: 10.3390/molecules26010223 (PMC7795100; doi:10.3390/molecules26010223)

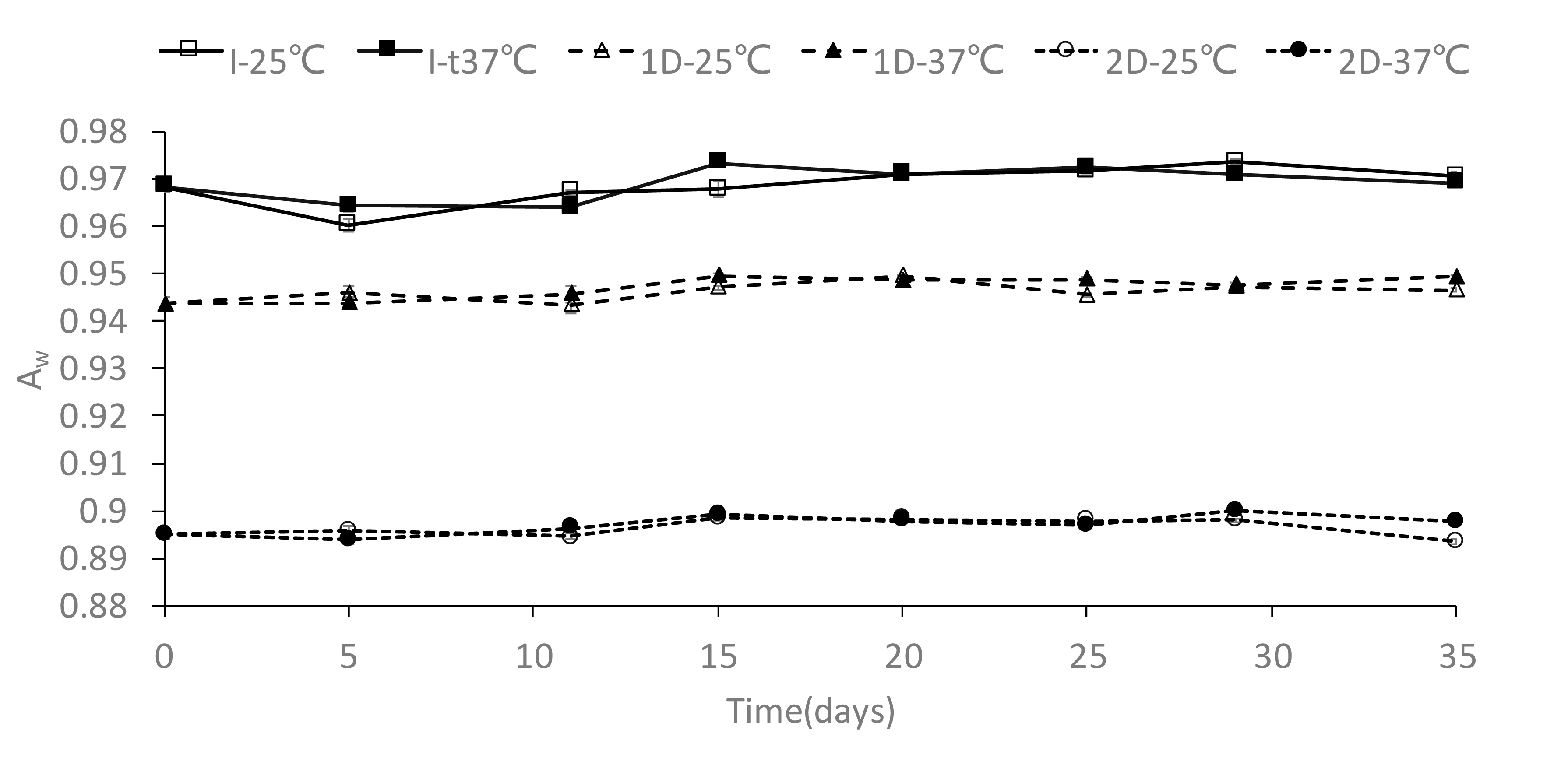

Supplement: Supplementary file 1 [file molecules-26-00223-s001.zip › Figure 1S.tif]

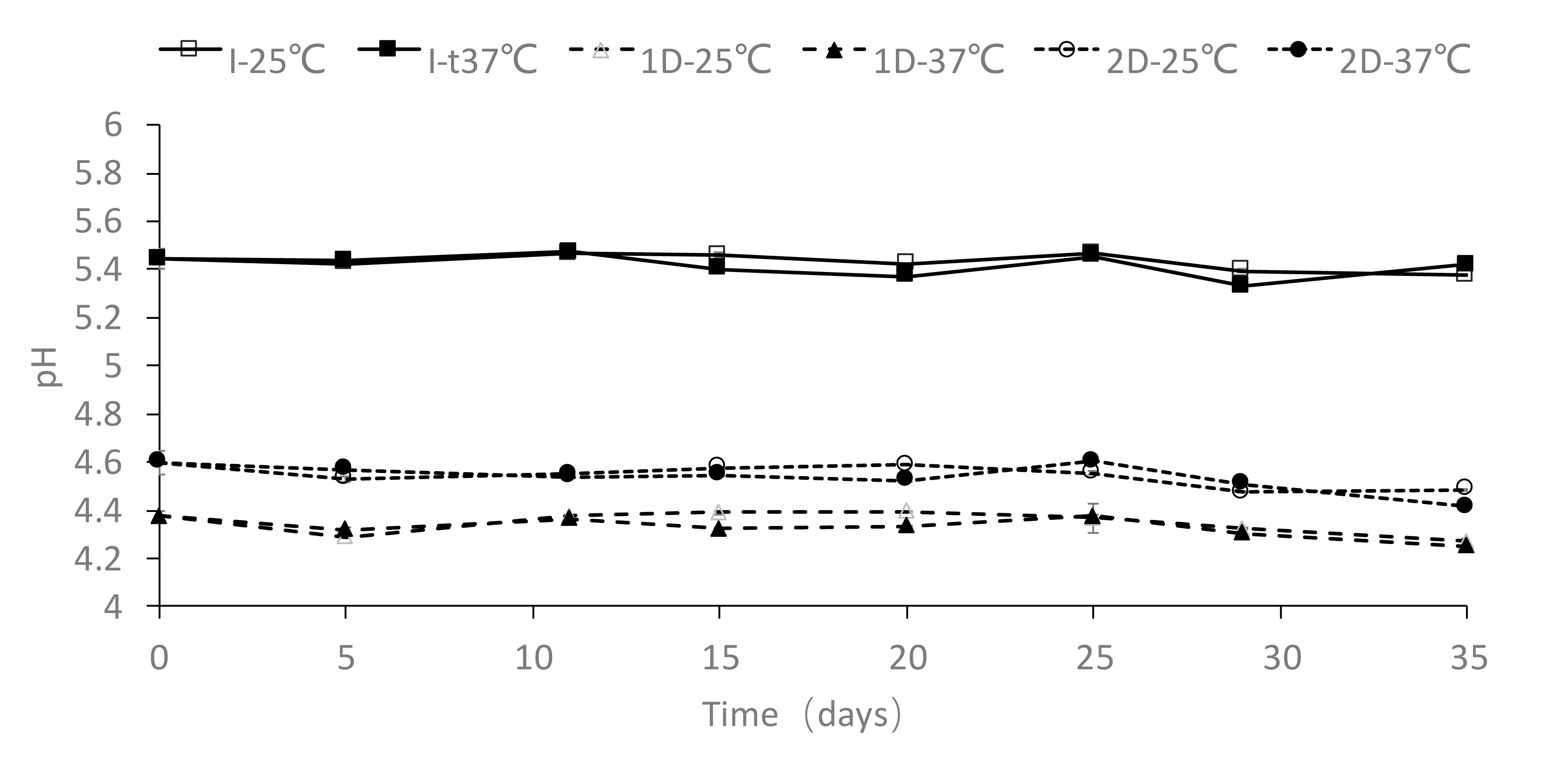

Supplement: Supplementary file 1 [file molecules-26-00223-s001.zip › Figure 2S.tif]
